# Characteristics of disk halo size and its correlation with lenticule quality in small incision lenticule extraction for moderate to high myopia

**DOI:** 10.1007/s00417-023-06133-x

**Published:** 2023-07-05

**Authors:** Yu Zhao, Wuxiao Zhao, Jifang Wang, Zhe Zhang, Xingtao Zhou, Jing Zhao

**Affiliations:** 1 Department of Ophthalmology and Optometry, Eye and ENT Hospital of Fudan University, 83 Fenyang Road, Shanghai, 200031 People’s Republic of China; 2 NHC Key Laboratory of Myopia (Fudan University); Laboratory of Myopia, Chinese Academy of Medical Sciences, Shanghai, China; 3 Shanghai Research Center of Ophthalmology and Optometry, Shanghai, China; 4 Center for Optometry and Visual Science, The People’s Hospital of Guangxi Zhuang Autonomous Region, Guangxi Academy of Medical Sciences, Nanning, China

**Keywords:** Disk halo size, Lenticule quality, Small incision lenticule extraction, Myopia

## Abstract

**Purpose:**

To investigate changes in disk halo size after small incision lenticule extraction (SMILE) and the correlation between halo size and lenticule quality in moderate to high myopia.

**Methods:**

Thirty eyes of 30 consecutive patients (mean age, 24.9 ± 4.5 years; mean spherical equivalent, −6.85 ± 1.18 D) undergoing SMILE were included in this prospective study. Lenticule surface quality was accessed with a scanning electron microscopy by a scoring system. Halo size was measured preoperatively and at 1, 3, and 6 months postoperatively. Multiple linear regression analysis was performed to explore associations between halo size and a range of factors, including lenticule quality.

**Results:**

Disk halo size increased slightly at 1 month and then recovered continually from 3 to 6 months postoperatively, with no difference between halo size during the preoperative period and at 6 months postoperatively (*P* > 0.05). One month after SMILE, halo size (1 cd/m^2^, 5 cd/m^2^) was associated only with uncorrected distance visual acuity (*P* ≤ 0.004). A halo size of 5 cd/m^2^ at 3 months postoperatively correlated with the anterior surface quality of the lenticule (*P* = 0.046). At 6 months postoperatively, a halo size of 1 cd/m^2^ was associated only with the baseline, accounting for 11.9% of the variability (*P* = 0.041); no correlations were found for the halo size of 5 cd/m^2^.

**Conclusions:**

Disk halo size after SMILE was enlarged at an early stage postoperatively and subsequently declined to the baseline level during a 6-month follow-up. The quality of the lenticule surface influenced halo size changes in the early phase.

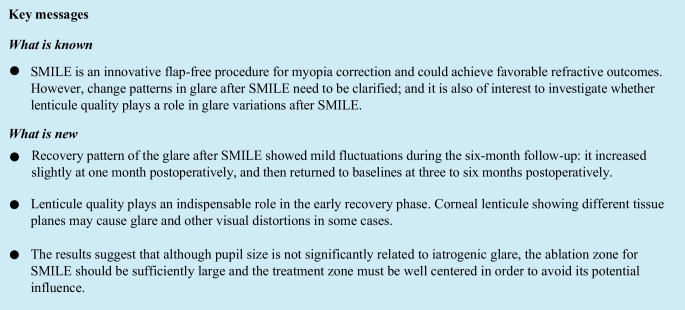

**Supplementary Information:**

The online version contains supplementary material available at 10.1007/s00417-023-06133-x.

## Introduction

Small incision lenticule extraction (SMILE) is an innovative procedure for myopia correction, where, the lenticule is scanned using a femtosecond laser and then manually extracted using a spatula and forceps [1]. Because smooth lenticule quality is vital to achieving fine visual acuity and overall visual quality, investigations have studied the characteristics of femtosecond laser settings, leading to an evolution within this subfield of ophthalmology [2–4]. For instance, it has been proposed that pulse energy and laser frequency are two vital parameters affecting lenticule quality and postoperative visual quality [4]. Most of the articles investigating this issue had suggested that high frequency and low energy settings allow for both finer cutting of corneal tissue and optimizing visual satisfaction [2]. Thus, high-frequency, low-energy femtosecond lasers are generally recommended for SMILE [5]. It should be noted that beyond that, the personalized settings are also needed when individual patient factors taken into considered. Glare is a symptom caused by forward-scattered light in the eyes with cataract, following corneal refractive surgery, cataract surgery or other eye procedures [6]. It affects patient satisfaction and causes visual disturbance, such as night driving or recognizing objects under intense light source. Previous studies focusing on glare following excimer laser refractive surgery have proposed that a large disk halo size could be induced by this type of surgery during the early postoperative period, but the impact would be short-lived and glare would be relieved in the long-term [6–8].

However, change patterns in glare after SMILE may differ from those after excimer laser surgery, and thus, need to be clarified within rigorous clinical research specific to this procedure. Additionally, it is of interest to investigate whether lenticule quality plays a role in glare variations after SMILE.

To address this gap, our study was aimed to evaluate the time course of glare after SMILE, as well as to investigate lenticule quality and other factors that may contribute to this issue.

## Patients and methods

### Study and patients

This prospective study consecutively recruited 30 eyes in 30 patients with myopia or myopic astigmatism (5 males and 25 females) aged 24.9 ± 4.5 years (18–35 years) who underwent SMILE surgery. All patients underwent routine preoperative examinations to exclude surgical contraindications. The preoperative corrected distance visual acuity (CDVA) was 20/20 or better in all patients, and no patients manifested ocular or systemic conditions. This research adhered to the tenets of the Declaration of Helsinki and was approved by the Institutional Review Board of the Eye & ENT Hospital of the Fudan University (registration number: ChiCTR1800017594). All participants provided written informed consent prior to participation.

Follow-up was scheduled at 1, 3, and 6 months postoperatively.

### Glare and halo test

As described previously [9], patients were tested unilaterally at a viewing distance of 2.5 m from the monitor (MonPack One, Metrovision, France) after 5 min of dark adaptation. Luminance conditions were consecutively set to 1, 5, and 100 cd/m^2^. Patients were tested for preoperative CDVA and postoperative UDVA. The light source was generated briefly and optotypes (arranged in three radical lines) were presented from the periphery towards the light source. Patients were instructed to read out the optotypes sequentially starting from the periphery. The unrecognized optotypes of each line were calculated in arcmin; these values were then averaged and treated as glare (disk halo size).

### SMILE procedure

The same surgeon (XZ) performed all procedures. As previously described [8], SMILE surgery was performed using a 500 kHz VisuMax^®^ platform (Carl Zeiss Meditec AG, Jena, Germany) with cap thickness of 120 μm, cap diameter of 7.5 mm, side cut length of 2 mm at the 12:00 position, optical zone of 6.1 to 6.9 mm, and calculated lenticule thickness of 102 to 150 μm. One lenticule was randomly selected from each patient for further surface quality examination. All lenticules were immediately stored for sample preparation. After surgery, 0.5% levofloxacin eye drops, 0.1% fluorometholone eye drops, and artificial tears were routinely prescribed for all patients.

### Sample preparation and surface quality index

Each lenticule was placed on a filter paper and marked with the anterior surface facing up. The lenticule was immediately immersed in 3% glutaraldehyde, 100 mM HEPES (N-2-hydroxyethylpiperazine-N′-2-ethanesulfonic acid; pH 7.4), 1 mM CaCl_2_, 1 mM MgCl_2_, and 25 mM NaN_3_ at 24 °C for 2 h, followed by overnight fixation at 4 °C. The phosphate buffer (0.1M) was rinsed twice (10 min per rinse), and distilled water rinsed once for another 10 min. The lenticule tissue was then transferred to a carbonization cabin for fumigation for ≥ 2 h. The carbonized cabin was filled with 600 μl fuel liquid (1% osmium tetroxide). After carbonization, the lenticule samples were transferred to an oven set to 40 °C for 1 h. Each lenticule was then cut into two pieces and mounted on scanning electron microscope (SEM) aluminum stubs, with one upward and the other downward. Samples were placed into the vacuum evaporator for coating with gold spray (EM AG600, Leica). The stubs automatically rotated by an electric current of 15 mA. The machine shut off automatically after detecting the specimen coated with a 10 nm gold. The specimen was imaged with SEM after a complete coating with gold spray (GeminiSEM 300, Zeiss). A scoring system was used to assess the surface characteristics of the evaluated lenticules. [5] Specifically, four criteria were used to assess surface morphology (Table 1). The surface relief was analyzed at a 100× magnification, and the three other criteria were evaluated at a 300× magnification. A maximum of 16 points was assigned to each lenticule. Two masked observers (YZ and XZ) graded the lenticules in random order.

**Table 1:** Criteria for evaluating surface characteristics

	Criterion and magnification	Appearance	Scores
A	Surface relief ×100	Very smooth	4
		Smooth	3
		Rough	2
		Very rough	1
B	Regularity of surface structure ×300	Completely regular	4
		Almost regular	3
		Partially regular	2
		Not regular	1
C	Portion of surface irregular ×300	< 10% of cut surface	4
		11–25% of cut surface	3
		26–50% of cut surface	2
		> 50% of cut surface	1
D	Position of the irregular area ×300	No irregularities	4
		Peripheral only	3
		Large region	2
		All over	1

### Statistical analyses

Descriptive data were expressed as means ± standard deviations. The Kolmogorov–Smirnov normality test and tests for homogeneity of variances were performed for all data. We performed analysis of variance (ANOVA) for repeated measures with Bonferroni correction to evaluate variations in halo size at different follow-up times. If the data were not suitable for ANOVA, Friedman’s rank test for k correlated samples was implemented instead. A stepwise multiple linear regression analysis was performed to explore possible factors affecting postoperative glare, such as lenticule quality, preoperative data, surgical parameters, and postoperative UDVA. SPSS statistical software (v.24, IBM Corp., Armonk, NY, USA) was used to conduct statistical analyses. Statistical significance was set to *P* < 0.05.

## Results

The preoperative data were as follows: sphere, −6.29 ± 1.12 D (−8.25 D to −3.00 D); astigmatism level, −1.35 ± 0.67 D (−2.75 D to 0 D); spherical equivalent, −6.85 ± 1.18 D (−9.00 D to −4.00 D); and CDVA, −0.02 ± 0.04 LogMAR (logarithm of the minimum angle of resolution; −0.1 to 0 LogMAR). The mean mesopic pupil size was 6.96 ± 0.46 mm (6.0 to 8.0 mm).

### Visual and refractive outcomes

**Fig. 1:**
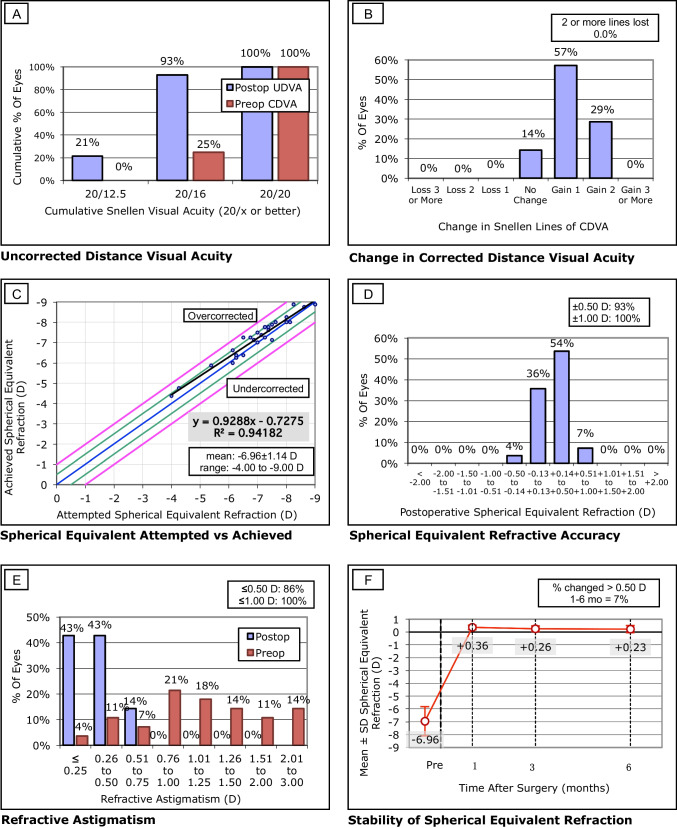
Refractive outcomes for patients after small incision lenticule extraction (SMILE). Postoperative cumulative uncorrected distance visual acuity (UDVA) after SMILE (**A**). Postoperative changes in Snellen lines of CDVA (**B**). Attempted versus achieved spherical equivalent refraction (**C**). Accuracy of spherical equivalent refraction (**D**). Postoperative refractive astigmatism (**E**). Stability of spherical equivalent refraction (**F**)

All surgeries were uneventful with no postoperative complications. Six months postoperatively, the mean safety index was 1.28 ± 0.17, and the mean efficacy index was 1.21 ± 0.17. All operated eyes had a postoperative UDVA of 20/20 or better (Fig. 1A), and no eyes lost one or more lines of CDVA (Fig. 1B). A scatter plot of the attempted versus the achieved spherical equivalent (SE) correction is shown in Fig. 1C. The mean SE was 0.25 ± 0.28 D, and 90% of the eyes were within ± 0.50 D; moreover, 100% of the eyes were within ± 1.00 D (Fig. 1D). The mean astigmatism was −0.38 ± 0.23 D and 100% of the eyes were within ± 0.50 D (Fig. 1E); between 1 and 6 months postoperatively, 7% of the evaluated eyes showed some level of change (Fig. 1F).

### Lenticule quality

Two lenticules (Nos.19 and 22) were seriously contaminated in preparation for imaging and a total of 56 samples (28 lenticules) were ultimately scored (Supplementary Table 1). Of the 28 lenticules, four (Nos. 10, 16, 17, and 30) showed excellent surface quality (i.e., with a total score of 16 points); Twenty-one lenticules had a score of ≥ 14 points. Among seven lenticules scored < 14 points: one case each scored 12 (No. 3) and 13 points (No. 6); No. 29 had the lowest score, with an anterior surface score of 11 and posterior score of 9; the remaining four samples each scored 13.5 points.

Twelve lenticules showed differently ordered residual tissue fibers. Therefore, all the lenticules were assigned to two groups based on “different tissue plane” and “same tissue plane” presentation. No significant differences were detected in the anterior surface (*P* = 0.617), posterior surface (*P* = 0.240), or in averaged surface scores (*P* = 0.622) between the evaluated groups (Table 2).

**Table 2:** Comparison of lenticule scores and glare between different and same tissue plane groups

	different tissue plane	same tissue plane	*P*
Anterior surface	14.58 ± 1.32	14.31 ± 1.26	0.617
Posterior surface	13.75 ± 1.83	14.56 ± 1.17	0.240
Average score	14.17 ± 1.42	14.44 ± 1.12	0.622
Preoperative, (arc min, 1 cd/m^2^)	206.67 ± 25.93	205.63 ± 43.58	0.931
Preoperative, (arc min, 5 cd/m^2^)	79.17 ± 12.56	81.25 ± 19.00	0.603
Postoperative-1m, (arc min, 1 cd/m^2^)	227.50 ± 49.35	216.25 ± 38.55	0.733
Postoperative-1m, (arc min, 5 cd/m^2^)	91.67 ± 24.44	85.00 ± 17.32	0.763
Postoperative-3m, (arc min, 1 cd/m^2^)	202.50 ± 28.61	198.75 ± 43.28	0.885
Postoperative-3m, (arc min, 5 cd/m^2^)	81.67 ± 13.44	76.88 ± 14.46	0.410
Postoperative-6m, (arc min, 1 cd/m^2^)	193.33 ± 45.34	191.88 ± 28.77	0.917
Postoperative-6m, (arc min, 5 cd/m^2^)	70.83 ± 14.41	73.13 ± 12.61	0.645

*m* month

The mean scores of the evaluated anterior, posterior, and combined surfaces were 14.43 ± 0.25, 14.21 ± 0.30, and 14.32 ± 0.24, respectively. There was no significant difference between the anterior and posterior surface scores; the surface quality of both sides was strongly correlated (*r* = 0.435, *P* = 0.021).

### Disk halo size

Results regarding glare under various luminance conditions and at different follow-up times are summarized in Table 3. At 100 cd/m^2^, all patients reported a halo size of 60 arcmin at all follow-up times. The halo size (at 1 cd/m^2^ and 5 cd/m^2^) increased slightly at 1 month postoperatively as compared to baselines and then decreased continually from 3 to 6 months postoperatively. At 6 months after SMILE, the mean halo size at 1 cd/m^2^ and 5 cd/m^2^ were 190.67 ± 37.04 and 72.00 ± 13.49, respectively. Although no significant differences were noted, findings at both luminance levels were smaller than baselines (203.33 ± 37.90 at 1 cd/m^2^ and 79.00 ± 17.09 at 5 cd/m^2^). Significant differences were found when comparing values at 1 month and 6 months postoperatively.

**Table 3:** Disk halo size under various luminance conditions before and after SMILE

		Time points				*P* value						
		Preop	Postop 1 month	Postop 3 months	Postop 6 months		Preop -1m	Preop -3m	Preop -6m	1m -3m	1m -6m	3m -6m
1 cd/m^2^	Average	203.33	216.67	197.67	190.67	0.015^†^	1.000	1.000	0.577	0.148	0.007	1.000
	SD	37.90	46.26	38.57	37.04							
5 cd/m^2^	Average	79.00	86.00	78.00	72.00	0.019^‡^	1.000	1.000	0.386	1.000	0.036	0.969
	SD	17.09	21.75	14.48	13.49							
100 cd/m^2^	Value	60	60	60	60	N/A						

*SMILE* small incision lenticule extraction, *preop* preoperative, *postop* postoperative, *m* month

^†^The analysis of variance for repeated measures (ANOVA) with the Bonferroni correction. ^‡^The Friedman’s Rank test for k correlated samples

### Regression analyses

Non-independent and non-correlated variables were excluded from multiple linear regression analyses using stepwise regression. Preoperatively, no association was detected between mesopic pupil or other baseline characteristics with respect to glare. One month postoperatively, halo size (at 1 cd/m^2^ and 5 cd/m^2^) was associated only with UDVA (*P* ≤ 0.004). At 3 months postoperatively, halo size (at 5 cd/m^2^) was correlated with anterior surface quality (*P* = 0.046), accounting for 12.0% of the glare variations; however, with respect to luminance level (at 1 cd/m^2^), only the preoperative halo size was found to be associated with data at the 3-month postoperatively (*P* = 0.002). At 6 months postoperatively, halo size (at 1 cd/m^2^) was only found to be associated with preoperative data, accounting for 11.9% of the glare variations (*P* = 0.041); no correlated factors were observed with respect to the halo size at 5 cd/m^2^. Detailed results of the linear regression analysis are presented in Table 4.

**Table 4:** The stepwise multiple linear regression model analysis for predicting glare after SMILE at different follow-up times

Value	Main predictors	B	SE	β	t	Sig.	Adjusted R^2^	F	Sig.
1 cd/m^2^ (pre)				N/A					
5 cd/m^2^ (pre)				N/A					
1 cd/m^2^ (post 1m)	Constant	403.809	52.738		7.657	< 0.001	0.294	12.228	0.002
	UDVA (post 1m)	−154.581	44.207	−0.566	−3.497	0.002			
5 cd/m^2^ (post 1m)	Constant	169.946	25.838		6.577	< 0.001	0.256	10.279	0.004
	UDVA (post 1m)	−69.440	21.659	−0.532	−3.206	0.004			
1 cd/m^2^ (post 3m)	Constant	84.436	34.860		2.422	0.023	0.278	11.416	0.002
	1 cd/m^2^ (pre)	0.563	0.166	0.552	3.379	0.002			
5 cd/m^2^ (post 3m)	Constant	149.077	34.042		4.379	< 0.001	0.120	4.423	0.046
	Anterior surface score	−4.923	2.341	−0.394	−2.103	0.046			
1 cd/m^2^ (post 6m)	Constant	112.815	37.553		3.004	0.006	0.119	4.648	0.041
	1 cd/m^2^ (pre)	0.387	0.179	0.389	2.156	0.041			
5 cd/m^2^ (post 6m)				N/A					

*B* unstandarized coefficients, *SE* standard error of unstandardized coefficients, *β* standardized coefficients (beta), *t* unstandardized coefficients/standard error, *Sig*. significance, *pre* preoperative, *post* postoperative, *m* month

## Discussion

With the refinement of the SMILE, it is necessary to build clinical findings regarding the factors responsible for glare to minimize postoperative patient complaints. Here, we investigated changes in glare after SMILE and determined the relationship between glare and lenticule quality and other critical factors.

The results revealed that the recovery pattern of the glare after SMILE showed mild fluctuations during the 6-month follow-up. Although it increased slightly at 1 month postoperatively (and then returned to baselines at 3 to 6 months postoperatively), the mean halo size observed was found to be less than that seen within the preoperative data during postoperative follow-up. Such phenomena have also been observed in previous studies investigating changes in glare after laser in situ keratomileusis (LASIK). Lackner et al. found that glare peaked 1 month postoperatively and subsequently decreased. At 6 months postoperatively, glare and halo still 1.74 times the preoperative value. However, it is worth mentioned that the sample size in the study was relatively small and may affect the accuracy of the results [6]. Another study evaluating glare after LASIK similarly demonstrated that symptoms were most severe 1 month postoperatively and declined continuously thereafter [10].

Apart from these results, studies on changes in glare after femtosecond laser refractive surgery have demonstrated similar results [7, 11, 12]. A study of nighttime symptoms in SMILE-treated eyes were compared to those of unoperated eyes for the same patients, and no differences were noted in terms of halo size [9]. Moreover, Han et al. investigated disk halo size (at 5 cd/m^2^) at 1 week and 3 months after SMILE and reported that SMILE induced a significant increase in halo size at 1 week postoperatively but that no change was observed at 3 months postoperatively [13]. Notably, the current study had a longer follow-up time and was also innovative in that it evaluated disk halo size changes at 1 cd/m^2^. 1 cd/m2 is the light condition darker than 5cd/m2, which could provide more information on patients’ visual symptoms at night. However, our results were consistent with the aforementioned studies. Our findings have illustrated the general rule shown in the literature to date regarding visual quality recovery after SMILE. Specifically, this procedure is expected to cause visual disturbances in early-stage, but the symptoms generally improve with time. The findings in these studies including ours suggested that an assessment of preoperative pupil sizes could be useful in identifying patients who may be at risk of declines in visual performance postoperatively.

In our study, multivariate linear regression analysis showed that glare at 1 month postoperatively was associated only with UDVA at both evaluated luminance levels. Moreover, in a large retrospective case series of LASIK patients, glare strongly associated with UDVA at 1 month postoperatively [14]. A relationship between glare and UDVA 3 months after LASIK was also observed in another prospective study [7]. However, it was not observed at 3 months in our study. Potential explanations for this discrepancy in changes in glare include differences in wounding healing response. For example, a comparative study led to the hypothesis that SMILE induces a lower degree of keratocyte apoptosis and proliferation and a lower inflammatory response when compared with LASIK [15]. As SMILE has several advantages over LASIK (i.e., the femtosecond laser allows for less severe tissue injury as compared with the excimer laser, and SMILE does not create a flap), it is inferred that patients undergoing SMILE might have a faster visual recovery than those undergoing LASIK in the early postoperative stage. Xu investigated the characteristics of forward light scatter changes after SMILE and femto-LASIK, the results showed a significant increase in the femto-LASIK group at 1 month after the procedure, whereas no significant increases after SMILE compared with baseline data; the percentage of increased stray light values in the SMILE group was lower than that in the femto-LASIK group [16]. More studies evaluating corneal healing response and its relation with visual recovery pattern are needed to support this hypothesis.

**Fig. 2:**
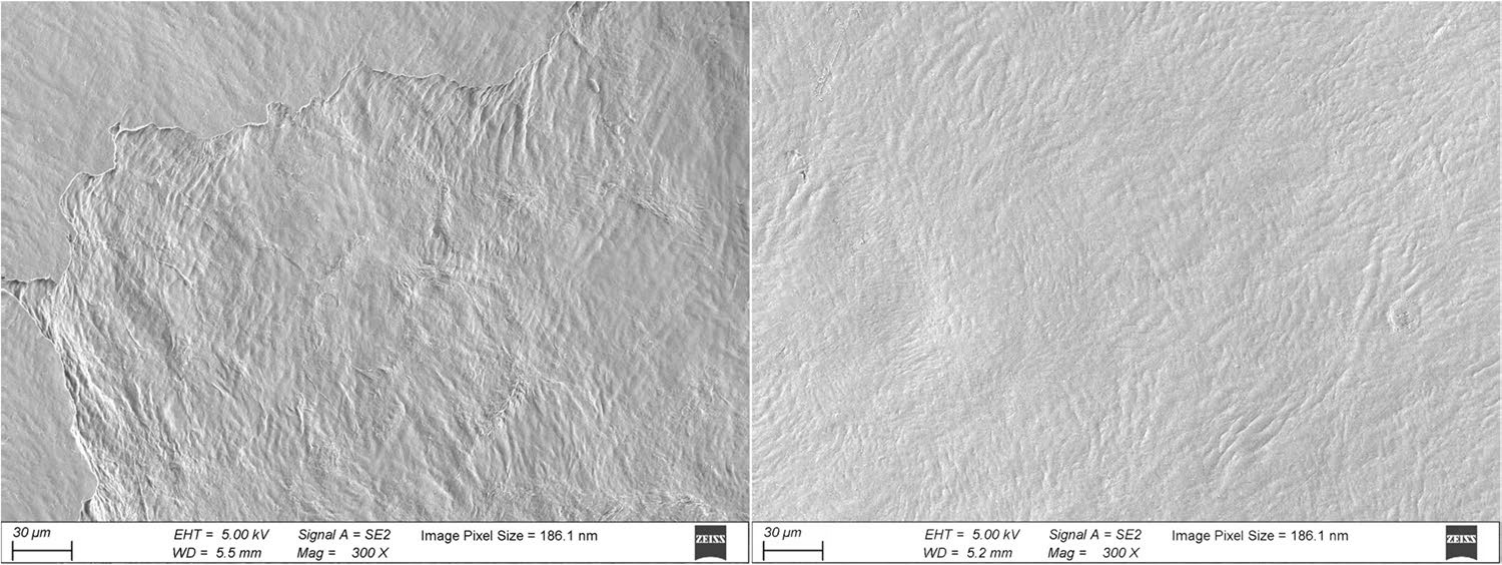
Residual tissue planes were identified by scanning electron microscope. Differently ordered residual tissue fibers were exhibited in “different tissue plane” group (300× magnification, left), and well-ordered residual tissue fibers were presented in “same tissue plane” group (300× magnification, right)

Regarding lenticule quality and glare, we only detected a significant relationship between anterior surface quality and halo size (at 5 cd/m^2^) 3 months postoperatively (*P* = 0.046); this finding explained 12% of the variability. Our results indicate that lenticule quality plays an indispensable role in the early recovery phase. It is worth noting that, in our study, 12 lenticules exhibited two differently ordered residual tissue fibers in SEM evaluation (Fig. 2). Among the lenticules that displayed differently ordered collagen fibers, three lenticules occurred on the anterior surface, whereas the other nine lenticules occurred on the posterior surface. The latter phenomenon can be explained by the working principle of femtosecond lasers. In the SMILE procedure, once the patient fixates on the target light and the centration is confirmed, the cornea is suctioned under the contact glass. As the contact glass curvature is flatter than the cornea, the cornea is applanated through the laser-scanning phase. Hence, scanning may occur in different stroma lamellae planes. Moreover, the existence of an opaque bubble layer and surgical manipulation may also cause residual corneal collagen fibers to be arranged in different minor lamellae planes.

The posterior part of the cornea was altered more than the anterior part in this procedure. Thus, complications associated with adverse outcomes regarding disk halo size are more likely to be distributed on the posterior lenticule surface. In this study, different residual tissue planes could be identified by SEM but this differing pattern did not lead to a significant increase in surface irregularity (Table 4). To investigate the impact of scanning quality on glare more comprehensively, we divided the evaluated eyes into two groups: “different tissue plane” and “same tissue plane” groups. Our results showed no significant differences under various luminance conditions between the two groups.

Interestingly, two patients had lenticule scores of less than 13 points (Nos. 3 and 29); these patients reported some differences in terms of changes in disk halo size after SMILE. Case 3 was assigned to the “same tissue plane” group, with a quality score of 12 points. Although the patient’s halo size at 1 cd/m^2^ increased from 200 arcmin at baseline to 260 arcmin at 1 month postoperatively, the halo size had recovered rapidly by 3 months postoperatively. At the final visit, the glare was similar to that in baselines (200 arcmin vs. 200 arcmin at 1 cd/m^2^, 80 arcmin vs. 70 arcmin at 5 cd/m^2^). Conversely, case 29 was assigned to the “different tissue plane” group and had a lenticule quality score of 10 points. This was the lowest score among all evaluated lenticules. The glare at 6 months postoperatively was larger than the baselines (270 arcmin vs. 210 arcmin at 1 cd/m^2^, 100 arcmin vs. 60 arcmin at 5 cd/m^2^). These two cases illustrate that, although different tissue planes do not induce statistically significance lenticule surface roughness, this presentation could cause glare and other visual distortions in cases with frequently occurring complications, such as inappropriate suction or decentration scanning.

It has been described that excessive surgical manipulation, cornea edema and eye rotation during the laser scanning phase are potential risk factors causing lenticule roughness [5, 17]. Thus good cooperation, short centration time and gentle surgical manipulation are essential in guaranteeing smooth lenticule surface quality.

At different time points after SMILE, we found that mesopic pupil size did not correlate with disk halo size (at 1 cd/m^2^ or 5 cd/m^2^). Whether a large mesopic pupil size leads to severe glare following refractive surgery is being debated [18]. Some researchers reported that large mesopic pupil size directly affected postoperative visual quality at night [19]. Conversely, other studies raised controversies regarding this view and have further speculated that this relationship may not exist [10, 14]. According to Villa et al., although pupil size is not significantly related to iatrogenic glare, the ablation zone for LASIK should be sufficiently large and the treatment zone must be well centered in order to avoid its potential influence [20]. We support this view and suggest measuring low-light pupil diameter prior to the surgery. Also, the above-mentioned criteria should likewise be considered while administering SMILE to maintain optimal visual quality.

This study has several limitations. The sample size was modest and a questionnaire was not included. Besides, a comparative team of patients treated with the same technique using a different laser setting would provide more powerful results. Future studies, such as those administering a subjective questionnaire within a larger database, are warranted. Despite the simplicity and somewhat limited results of our findings, this preliminary study provides valuable information for understanding glare after SMILE, which can guide future research and directly inform medical guidelines.

In conclusion, glare was enlarged at early-stage after SMILE and subsequently declined to the baselines during the 6-month follow-up. Lenticule surface quality was found to significantly influence glare in the early-stage postoperatively.

### Supplementary Information

Below is the link to the electronic supplementary material.

Supplementary file1 (DOCX 51.3 KB)

## Data Availability

The authors confirm that the data supporting the findings of this study are available within the article and its supplementary materials.
