# Supplementary material for: Characteristics of disk halo size and its correlation with lenticule quality in small incision lenticule extraction for moderate to high myopia
Source: Graefes Arch Clin Exp Ophthalmol. 2023 Jul 5;262(1):313–21. doi: 10.1007/s00417-023-06133-x (PMC10805804; doi:10.1007/s00417-023-06133-x)
Supplement: Supplementary file 1 — Supplementary file1 (DOCX 51.3 KB) [file 417_2023_6133_MOESM1_ESM.docx]

Supplementary Table 1. Score results for all lenticules.

|  |  |  |  |  |  |  |  |  |  |  |  |  |  |  |  | Average | SD |
| --- | --- | --- | --- | --- | --- | --- | --- | --- | --- | --- | --- | --- | --- | --- | --- | --- | --- |
| Anterior  Surface | 16 | 16 | 14 | 15 | 16 | 15 | 14 | 16 | 14 | 14 | 15 | 12 | 13 | 12 | 11 | 14.43 | 0.25 |
| Posterior  Surface | 16 | 15 | 16 | 15 | 14 | 14 | 15 | 13 | 14 | 13 | 12 | 15 | 13 | 12 | 9 | 14.21 | 0.30 |
| Average | 16 | 15.5 | 15.5 | 15 | 15 | 14.5 | 14.5 | 14.5 | 14 | 13.5 | 13.5 | 13.5 | 13 | 12 | 10 | 14.32 | 0.24 |
| Cases | 10,16  17,30 | 5 | 12,21 | 7,2324 | 20 | 8,14 | 15,27 | 18 | 2,4,1325,26 | 9,28 | 11 | 1 | 6 | 3 | 29 |  |  |

SD = *standard deviation*
